# Artificial gauge field switching using orbital angular momentum modes in optical waveguides

**DOI:** 10.1038/s41377-020-00385-6

**Published:** 2020-08-28

**Authors:** Christina Jörg, Gerard Queraltó, Mark Kremer, Gerard Pelegrí, Julian Schulz, Alexander Szameit, Georg von Freymann, Jordi Mompart, Verònica Ahufinger

**Affiliations:** 1 Physics Department and Research Center OPTIMAS, Technische Universität Kaiserslautern, 67663 Kaiserslautern, Germany; 2 Departament de Física, Universitat Auto’noma de Barcelona, E-08193 Bellaterra, Spain; 3 Institut für Physik, Universität Rostock, Albert-Einstein-Straße 23, 18059 Rostock, Germany; 4 Department of Physics and SUPA, University of Strathclyde, Glasgow, G4 0NG UK; 5 Fraunhofer Institute for Industrial Mathematics ITWM, 67663 Kaiserslautern, Germany

**Keywords:** Integrated optics, Photonic devices

## Abstract

The discovery of artificial gauge fields controlling the dynamics of uncharged particles that otherwise elude the influence of standard electromagnetic fields has revolutionised the field of quantum simulation. Hence, developing new techniques to induce these fields is essential to boost quantum simulation of photonic structures. Here, we experimentally demonstrate the generation of an artificial gauge field in a photonic lattice by modifying the topological charge of a light beam, overcoming the need to modify the geometry along the evolution or impose external fields. In particular, we show that an effective magnetic flux naturally appears when a light beam carrying orbital angular momentum is injected into a waveguide lattice with a diamond chain configuration. To demonstrate the existence of this flux, we measure an effect that derives solely from the presence of a magnetic flux, the Aharonov-Bohm caging effect, which is a localisation phenomenon of wavepackets due to destructive interference. Therefore, we prove the possibility of switching on and off artificial gauge fields just by changing the topological charge of the input state, paving the way to accessing different topological regimes in a single structure, which represents an important step forward for optical quantum simulation.

During the last decade, the growing interest in quantum simulation has fostered the development of several techniques for implementing effective electromagnetic fields in systems of neutral particles^1,2^. In this vein, artificial gauge fields (AGFs) have been widely used in photonics to control light dynamics^3–5^, emulating the effect of electromagnetic fields on charged particles. Moreover, AGFs have also allowed the exploration of a plethora of phenomena stemming from their close connection to topological phases of matter^6–9^ (see Ozawa et al.^10^ for a recent review). Typically, these AGFs are introduced either by geometric manipulation^4,5^ or by time-dependent modulation^11–13^. While in Wu et al.^14^, wavepackets carrying orbital angular momentum (OAM) were used to create edge states in crystalline topological insulators, here, we experimentally demonstrate that an AGF in the form of an effective magnetic flux can be induced using Laguerre–Gauss light beams carrying OAM^15^. Specifically, to prove the existence of this flux, we show how Aharonov-Bohm (AB) caging naturally appears when OAM modes with a specific topological charge are injected into cylindrical optical waveguides arranged in a diamond chain configuration^16,17^.

AB caging, which was originally studied in the context of two-dimensional electronic systems, is a single-particle localisation effect arising from the interplay between the lattice geometry and a magnetic flux. More specifically, a constant magnetic flux modifies the phase relations of wavepackets, resulting in a destructive interference effect that binds the modes. Thus, it enables one to halt all propagation by controlling the flux. This phenomenon, which can be interpreted in terms of quantum interference^18,19^, has been predicted to occur^20–22^ and experimentally verified^23,24^ in photonic structures implementing AGFs. Unlike the previous photonic proposals based on geometric manipulation^20–22^, we show in this work how non-zero-energy flat bands, which are responsible for the caging effect, can be naturally and deliberately achieved by injecting light carrying OAM instead of fabricating a new sample. Therefore, our proposal enables the study of the effect of AGFs in photonic lattices just by selecting the topological charge of the input beam. In this context, our proposal differs from related works where the intrinsic angular momentum, i.e., the polarisation of the input beam, instead of the extrinsic one, i.e., the OAM, was used as the AGF switching mechanism^25^. Moreover, this method also allows access to different topological regimes without the need to fabricate different structures or employ high intensities, as is the case for topological phase transitions realised via non-linear optics^26^.

To experimentally visualise the AB caging effect induced by OAM modes, we fabricate photonic lattices composed of direct laser written optical waveguides^27^ arranged in a diamond chain configuration, as displayed in Fig. 1a. The unit cell *j* is composed of three waveguides $\left( {S_j = A_j,B_j,C_j} \right)$ forming a triangle with a central angle *θ*. Each cylindrical waveguide sustains OAM modes of the form^28^

1 $${\mathrm{\Psi }}_{S_j}^{ \pm \ell }\left( {r_{S_j},\phi _{S_j},z} \right) = \psi _{S_j}^\ell \left( {r_{S_j}} \right)e^{ \pm i\ell \left( {\phi _{S_j} - \phi _0} \right)}e^{ - i\beta _\ell z}$$

where $\ell = 0,1,2, \ldots$ is the topological charge, ± accounts for positive and negative circulation of the phase front, $\psi _{S_j}^\ell \left( {r_{S_j}} \right)$ is the radial mode profile given by the Bessel functions^15^, $(r_{S_j},\phi _{S_j})$ are the polar coordinates with respect to the centre of each waveguide *S*_*j*_ in the transverse plane, *z* is the propagation direction, *ϕ*_0_ is an arbitrary phase origin, and $\beta _\ell$ is the propagation constant of mode $\ell$. Moreover, while between fundamental modes $(\ell = 0)$, there is only one coupling amplitude $c_{0,0} \equiv c_0$, between OAM modes $(\ell \, \ne \, 0)$ with the same or opposite circulation directions, there are two coupling amplitudes $c_{\ell ,\ell } \equiv c_1$ and $c_{\ell , - \ell } \equiv c_2e^{i2\ell \phi _0}$^29^. In particular, as a proof of concept, we restrict our implementation to the $\ell = 0$ and $\ell = 1$ modes by properly engineering the refractive index contrast and the width of the step-index profile presented in Fig. 1b. In this case, between the $\ell = 1$ modes with the same or opposite circulation directions, there are two coupling amplitudes $c_{1,1} \equiv c_1$ and $c_{1, - 1} \equiv c_2e^{i2\ell \phi _0}$^29^. Therefore, when dealing with OAM modes, complex coupling amplitudes between modes with different circulation directions appear naturally. The different coupling strengths $c_0,\;c_1$ and *c*_2_ are presented in Fig. 1c (see Supplementary I for details on the calculations). Specifically, we set the phase origin *ϕ*_0_ along the $A_j \leftrightarrow C_j$ direction such that $c_{1, - 1} = c_2$ is real in this direction, while $c_{1, - 1} = c_2e^{ - i2\ell {\uptheta}}$ is complex along the $A_j \leftrightarrow B_j$ direction. In particular, we fix *θ* = *π*/*2*, which allows the coupling between modes propagating in next-nearest neighbour waveguides to be neglected^30^ (see Supplementary II for a detailed discussion). Moreover, for this specific angle, a relative phase difference of *π* between the $c_{1, - 1}$ couplings in the $A_j \leftrightarrow C_j$ and $A_j \leftrightarrow B_j$ directions appears. This phase difference introduces a *π* flux into the plaquettes that opens an energy gap between the dispersive bands, as discussed in detail in the following.

**Fig. 1:**
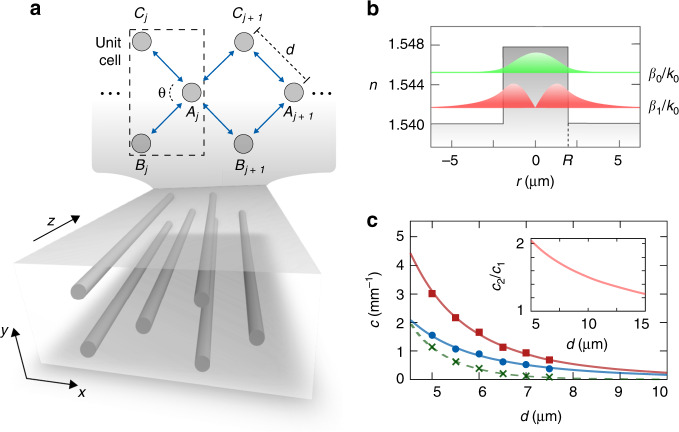
Lattice structure and optical waveguides. **a** Schematic representation of the structure composed of identical cylindrical waveguides arranged in a diamond chain configuration. Each unit cell *j* hosts three waveguides $s_j \equiv A_j,\;B_j,\;C_j$ forming a triangle with central angle *θ*. The distances between waveguide centres are $d_{A_j - B_j} = d_{A_j - C_j} \equiv d$, $d_{B_j - C_j} = 2d\sin (\theta /2)$ and $d_{A_j - A_{j + 1}} = 2d\cos (\theta /2)$. The blue arrows indicate the couplings. **b** Refractive index profile of the waveguides, defined by *n*_core_ = 1.548, *n*_clad_ = 1.540 and waveguide radius *R* = 1.9 μm. Field intensity of the $\ell = 0$ (green) and $\ell = 1$ (red) modes, where $\beta _\ell$ is the propagation constant of mode $\ell$, *k*_0_ = 2*π*/*λ*_0_ is the vacuum wavenumber and *λ*_0_ is the light wavelength in vacuum. **c** Numerically calculated coupling strengths for separation distances *d* = 5 μm, 5.5 μm, 6 μm, 6.5 μm, 7 μm and 7.5 μm using *λ*_0_ = 700 nm. In particular, *c*_0_ (crosses) accounts for the coupling between the $\ell = 0$ modes, and *c*_1_ (circles) and *c*_2_ (squares) account for the coupling between the $\ell = 1$ modes with the same or opposite circulation directions, respectively. The dashed and solid lines correspond to the exponential fittings of $c_0\left( d \right) \approx K_0\exp ( - \kappa _0d)$, $c_1(d) \approx K_1\exp ( - \kappa _1d)$ and $c_2(d) \approx K_2\exp ( - \kappa _2d)$, where *K*_0_ = 387 mm^−1^, *κ*_0_ = 1.17 μm^−1^, *K*_1_ = 19.39 mm^−1^, *κ*_1_ = 0.52 μm^−1^, *K*_2_ = 56.25 mm^−1^ and *κ*_2_ = 0.59 μm^−1^. The inset in **c** shows *c*_2_/*c*_1_ with respect to the separation distance *d*

Assuming periodic boundary conditions, the bulk band structure for the $\ell = 0$ modes consists of one flat and two dispersive bands (Fig. 2a), with energies given by^20^

2 $$E_0^0\left( k \right) = 0,\;E_ \pm ^0\left( k \right) = \pm 2c_0\sqrt {1 + {\mathrm{cos}}(k\sqrt 2 d)}$$

where *k* is the quasi-momentum and $\sqrt 2 d$ is the lattice constant. On the other hand, as presented in Fig. 2b, the band structure for $\ell = 1$ is composed of six energy bands, i.e., three bands with a twofold degeneracy (positive and negative circulation)^16^

3 $$E_0^1\left( k \right) = 0,\\E_ \pm ^1\left( k \right) = \pm 2\sqrt {\left( {c_1^2 + c_2^2} \right) + \left( {c_1^2 - c_2^2} \right){\mathrm{cos}}(k\sqrt 2 d)}$$

**Fig. 2:**
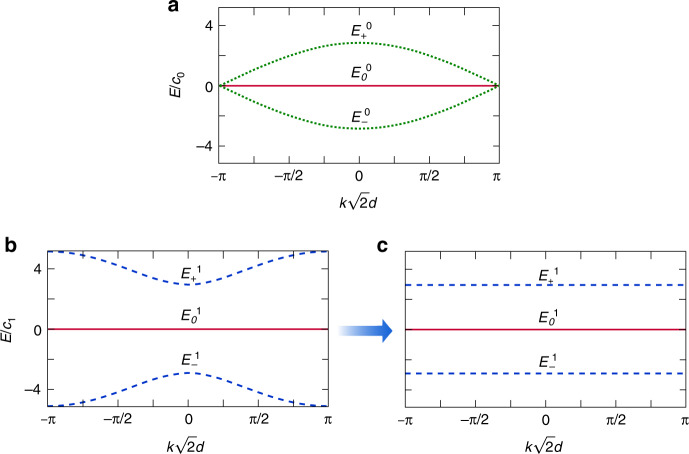
Energy band structure. **a** Band structure of the considered diamond chain lattice for $\ell = 0$ consisting of two dispersive bands $E_ - ^0(k)$ and $E_ + ^0(k)$ (dotted green lines) and one zero-energy flat band $E_0^0\left( k \right)$ (solid red line). Band structure of the considered diamond chain latti**c**e for $\ell = 1$ when **b** *c*_2_/*c*_1_ = 2 and **c** *c*_2_/*c*_1_ = 1. In **b** and **c**, each band has a two-fold degeneracy, i.e., $E_ - ^1(k) \equiv E_1\left( k \right) = E_2\left( k \right)$ (dashed line), $E_0^1(k) \equiv E_3\left( k \right) = E_4\left( k \right)$ (solid line) and $E_ + ^1(k) \equiv E_5\left( k \right) = E_6\left( k \right)$ (dashed line)

The main difference between the energy bands in the two cases is the existence of an energy gap for $\ell = 1$, which is absent for $\ell = 0$, indicating the presence of an AGF. By performing a basis rotation (Supplementary III), the original diamond chain can be decoupled into two identical chains with three energy bands and a *π* flux through the plaquettes that opens the energy gap^16^. Moreover, as illustrated in Fig. 2c, in the $c_2/c_1 \to 1$ limit, the dispersive bands $E_ \pm ^1 \to \pm 2\sqrt 2 c_1$ become flat, and the associated supermodes are localised in the $A_j,B_j,B_{j + 1},C_j$ and $C_{j + 1}$ waveguides. Therefore, if one excites *A*_*j*_ with a $\ell = 1$ mode, then the injected intensity will oscillate between the central and four surrounding waveguides, as predicted by the AB caging effect (Supplementary III).

To experimentally demonstrate AB caging using OAM modes, we excite a central waveguide *A*_*j*_ using modes with and without OAM and compare the resulting dynamics. We fabricate several samples with seven unit cells with different total lengths (ranging from *z* = 250 μm to *z* = 1000 μm) and extract the output pattern intensities. A scheme of the samples is depicted in Fig. 3. First, as displayed in Fig. 3a, we inject a mode with $\ell = 1$ and negative circulation into *A*_4_ (see Supplementary IV for complementary results). The injected mode spreads to the four surrounding waveguides at *z* = 250 μm (Fig. 3b) and recombines in the central waveguide at *z* = 500 μm (Fig. 3c). This spreading and recombination effect can be observed a second time at 750 μm (Fig. 3d) and 1000 μm (Fig. 3e). Even though we implement the model with $c_2/c_1 \approx 2$ due to experimental restrictions on the total size of the samples, we measure two full oscillations of the AB caging effect. Since the dispersive bands are not totally flat, light propagates into waveguides *A*_3_ and *A*_5_ during the second oscillation, and part of the intensity escapes from the cage (Fig. 3d, e). Additionally, although we try to excite the donut mode with negative circulation (see the input beam in Fig. 3a), the propagating mode has a lobe-shaped intensity (Fig. 3b–e) corresponding to a superposition of donut modes with positive and negative circulation. This lobe-shaped mode appears due to a slight ellipticity of the fabricated waveguides and the influence of the surrounding waveguides (see Supplementary IV for a complementary discussion). Nevertheless, since the propagation of the $\ell = 1$ modes with positive and negative circulation results in the same flux, the observed AB caging is the same for any superposition of both types of circulation, i.e., a lobe-shaped mode (Supplementary III). In contrast, the $\ell = 0$ mode injected into *A*_4_ only spreads transversally as it evolves along the propagation direction, and no caging is observed in Fig. 3f–j.

**Fig. 3:**
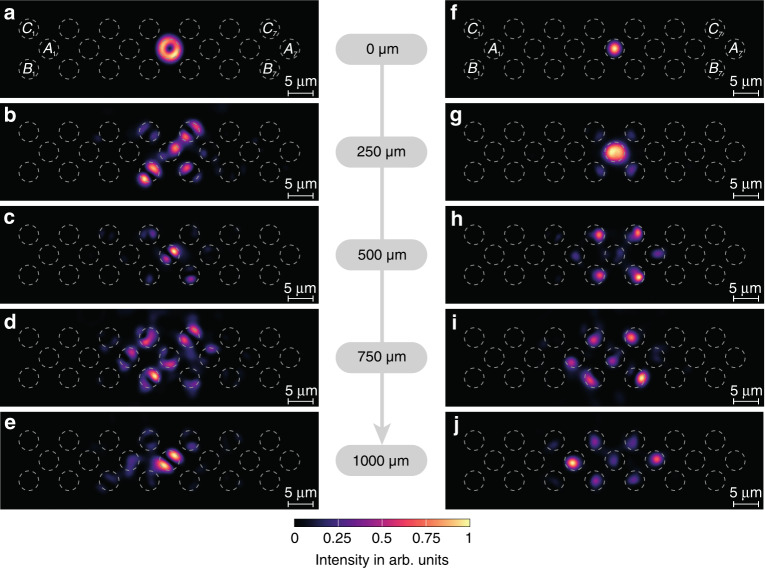
Aharonov-Bohm caging effect. Experimentally observed input and output intensities obtained by exciting the *A*_4_ waveguide using the OAM mode with $\ell = 1$ and negative circulation at **a** *z* = 0 μm, **b** *z* = 250 μm, **c** *z* = 500 μm, **d** *z* = 750 μm and **e** *z* = 1000 μm and with $\ell = 0$ at **f** *z* = 0 μm, **g** *z* = 250 μm, **h** *z* = 500 μm, **i** *z* = 750 μm and **j** *z* = 1000 μm. Note that the image in **a** is taken before entering the sample. The diamond chain lattice is composed of seven unit cells, i.e., 21 waveguides with radius *R* = 1.9 μm and nearest-neighbour separation *d* = 5.5 μm. The wavelength used is *λ*_0_ = 700 nm. The intensity distribution in each figure is normalised to the maximum intensity value of the corresponding figure

Finally, we compare the experimental observations of the light dynamics with numerical calculations. Figure 4 shows the intensity extracted at the output port from the *A*_4_ waveguide and its associated cage formed by $A_4,\;B_4,C_4,B_5,\;C_5$. In Fig. 4a, we can observe how the experimentally measured intensity maxima in *A*_4_ associated with the caging phenomenon occur around *z* = 500 μm and 1000 μm, in agreement with finite-difference method (FDM) simulations. On the other hand, in Fig. 4b, one can observe the standard decay of the intensity in *A*_4_ when the $\ell = 0$ mode is injected. Moreover, we also compute the light dynamics for longer distances using coupled-mode equations (Supplementary I and II). In Fig. 4c, one can observe how for $\ell = 1$, the first and second intensity maxima in *A*_4_ have ~60% and 10% of the injected intensity, respectively, which can be increased by reducing the difference between *c*_1_ and *c*_2_ (see inset of Fig. 1c). For example, for $c_2/c_1 \approx 1.25,$ i.e., *d* = 15 μm, the first and second maxima increase up to 97% and 80%, respectively, achieving 100% in the flat-band limit. However, larger separations between waveguides require longer samples, which were not feasible in our experiments. Alternatively, for $\ell = 0$, the intensity in *A*_4_ exponentially decays independent of the waveguide separation, confirming the different origins of the oscillations. Finally, note that the agreement between the experimental results obtained with different samples and waveguides and injecting modes with both types of circulation shown in Supplementary IV confirms the robustness of the AB caging effect since each measurement includes slight parameter variations (see Supplementary V for more details).

**Fig. 4:**
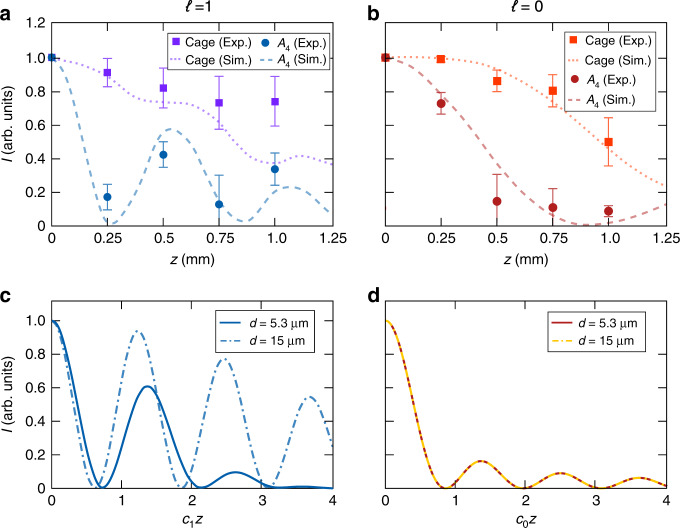
Light dynamics along the propagation direction. Intensity extracted from waveguide *A*_4_ (circles) and from the cage formed by $A_4,\;B_4,C_4,B_5,\;C_5$ (squares) normalised to the intensity extracted from the entire lattice as a function of the propagation distance *z* when the **a** $\ell = 1$ mode and **b** $\ell = 0$ mode is injected into waveguide *A*_4_. The results shown in **a** are an average of the intensities extracted for $\ell = 1$ with positive and negative circulation. The circles and squares correspond to the experimentally extracted intensities, while the lines correspond to the best-fitting curve of the simulated results obtained using FDM numerical techniques. The error bars associated with the experimental data are estimated taking into account a refractive index error of Δ*n* = ±0.001 in the fabrication process. Intensity propagating in waveguide *A*_4_ numerically calculated using coupled-mode equations as a function of *z* when the **c** $\ell = 1$ mode and **d** $\ell = 0$ mode is injected into waveguide *A*_4_. The solid lines correspond to the case with *d* = 5.3 μm, i.e., $c_2/c_1 \approx 2$, while the dashed lines correspond to *d* = 15 μm, i.e., $c_2/c_1 \approx 1.25$. Note that the simulations were performed considering *N* = 7 unit cells and *λ*_0_ = 700 nm with a correction of Δ*d* = –0.2 μm with respect to the expected experimental distance *d* = 5.5 μm. This difference may originate from slight variations in the position during the writing process (±0.05 μm) and small changes in the refractive index contrast

In summary, we demonstrated that an artificial gauge field in the form of an effective magnetic flux could be induced in a photonic lattice by exploiting the orbital angular momentum carried by light beams. Specifically, we demonstrated the appearance of this synthetic flux by experimentally measuring the photonic analogue of the Aharonov-Bohm caging effect for an arrangement of direct laser written cylindrical waveguides in a diamond chain configuration. Using this structure, we showed how an energy gap is opened between the dispersive bands of the system when light carrying OAM is injected, analogous to the effect produced by an artificial gauge field^20^. Moreover, we proved how non-zero-energy flat bands, which yield the AB caging effect, can be achieved by properly tuning the geometry of the unit cells and the separation between waveguides. The agreement between the dynamics revealed by the coupled-mode equations, the FDM simulations and the experiments confirms the validity of the presented model, which constitutes a step towards accessing different topological regimes in an active way by controlling the input states. Moreover, the inherently infinite dimensionality of OAM modes^15^ can be potentially exploited to increase the transmission capacity by using mode multiplexing^31^, paving the way towards combining integrated spatial multiplexing^32^ with topological protection^10^.

## Materials and methods

### Sample fabrication

The waveguide samples were fabricated via direct laser writing (DLW)^33^ using a commercial Nanoscribe system and the photoresist IP-Dip. To create waveguides in a single writing step, the inside of waveguides was written with higher laser power (60%) than the surrounding material (35%), which resulted in a refractive index contrast Δ*n* of ~0.008. The scan speed used was 20 mm/s. Multiple samples were fabricated (each on its own substrate) with different total lengths corresponding to *z* = 250 μm, 500 μm, 750 μm and 1000 μm. We used a waveguide radius of *R* = 1.9 μm and a centre-to-centre distance of *d* = 5.5 μm. In contrast to common methods, where the sample is placed in isopropanol after writing to remove the non-polymerised resist, here, the sample was not developed. Excess resist on the sample output facet was observed to distort the images during measurements. Therefore, this resist was removed by carefully dabbing the sample facet with a tissue wetted by isopropanol.

During the writing process, the laser intensity towards the edges of the sample decreased due to vignetting of the writing objective lens. At the same time, the proximity effect^34^ had less influence at the edges of the sample than in the centre. Both phenomena led to a non-uniform refractive index profile of the sample, with a higher index in the centre and a lower index at the edges. Preliminary results^27^ led us to assume that the index does not increase linearly with the used writing power but saturates for high powers below the threshold for resist destruction. As a result, the waveguides written with a high laser power were less prone to refractive index changes by vignetting and the proximity effect than the material surrounding the waveguides (written with a low laser power). The refractive index contrast between the waveguides and surrounding material is therefore supposed to increase towards the edges of the sample. Therefore, the measurements were performed on the central waveguides (*A*_3_ and *A*_4_).

### Measurement

The full setup can be seen in Supplementary VI.

Laser light from a white light laser (NKT photonics) was sent through a VARIA filter box to select a wavelength of 700 nm. The beam was linearly polarised, expanded and sent to a spatial light modulator (SLM). We loaded a hologram onto the SLM that consisted of a phase-only vortex, with an added blazed grating to shift the pattern to the first diffraction order. Other orders were blocked by a pinhole. The beam was circularly polarised and imaged onto an objective lens, which Fourier transformed the phase hologram to create a donut-shaped intensity profile with $\ell = 1$ and positive/negative circulation or a Gaussian-shaped intensity profile with $\ell = 0$ and constant phase (depending on the hologram that we loaded).

The reflection of the input mode was imaged via a beamsplitter onto camera 1. The use of white light from a common torch lamp allowed additional imaging of the sample input facet onto camera 1 at the same time to overlay the input mode with the waveguide position. The output intensity at the sample output facet was imaged onto camera 2. The intensity distributions for the different outputs were normalised to the maximum value to increase the visibility. Moreover, the recorded images were post-processed to reduce noise. This was achieved by overlaying the pictures with a mask of the waveguide structure at the position determined by a convolution. In this way, the intensities within the waveguides and in the surroundings were separated. The noise level of the surroundings was then subtracted from the original recorded picture. All resulting negative values were set to zero. To extract the intensities shown in Fig. 4, we subsequently integrated over a circle that covered almost the whole mode at the position of each waveguide. The circles were as large as possible such that they touched at the diagonals.

## Supplementary information

Supplementary information

## Data Availability

All experimental data and any related experimental background information not mentioned in the text are available from the authors upon reasonable request.
