# Supplementary material for: Artificial gauge field switching using orbital angular momentum modes in optical waveguides
Source: Light Sci Appl. 2020 Aug 28;9:150. doi: 10.1038/s41377-020-00385-6 (PMC7455748; doi:10.1038/s41377-020-00385-6)
Supplement: Supplementary file 1 — Supplementary information [file 41377_2020_385_MOESM1_ESM.docx]

-Supplementary information-

Artificial gauge field switching using orbital angular momentum modes in optical waveguides

Christina Jörg^1†^,Gerard Queraltó^2†*^, Mark Kremer^3^, Gerard Pelegrí^2,4^, Julian Schulz^1^, Alexander Szameit^3^, Georg von Freymann^1,5^, Jordi Mompart^2^, and Verònica Ahufinger ^2^

*^1^Physics Department and Research Center OPTIMAS, Technische Universität Kaiserslautern, 67663 Kaiserslautern, Germany*

*^2^Departament de F´ısica, Universitat Auto`noma de Barcelona, E-08193 Bellaterra, Spain*

*^3^Institut fu¨r Physik, Universita¨t Rostock, Albert-Einstein-Straße 23, 18059 Rostock, Germany*

*^4^Department of Physics and SUPA, University of Strathclyde, Glasgow G4 0NG, UK*

*^5^Fraunhofer Institute for Industrial Mathematics ITWM, 67663 Kaiserslautern, Germany*

^†^These authors contributed equally to this work.

*corresponding author gerard.queralto@uab.cat

This Supplementary information consists of the following sections:

1. **Unit cell structure and complex couplings for** $\mathcal{l=}\boldsymbol{1}$ **modes**
2. **Nearest neighbor vs. next-nearest neighbor couplings**
3. **Basis rotations for** $\mathcal{l=}\boldsymbol{1}$ **modes**
4. **Complementary results of the Aharonov-Bohm caging effect**
5. **Robustness of the Aharonov-Bohm caging effect**
6. **Experimental measurement set-up**

**Supplementary I: Unit cell structure and complex couplings for** $\mathcal{l=}\boldsymbol{1}$ **modes**

**
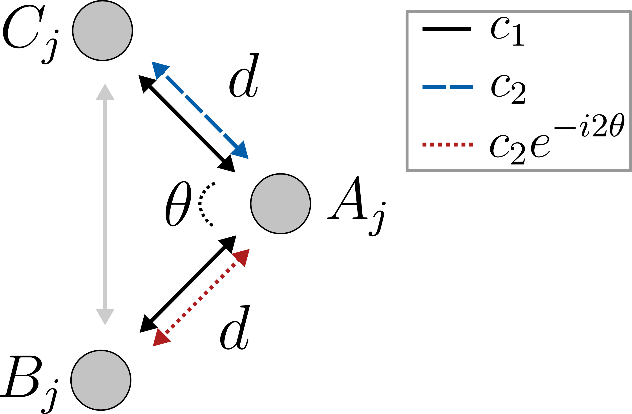
**

**Fig. S1. Unit cell structure and complex couplings for** $\mathcal{l=}\boldsymbol{1}$ **modes.** Schematic representation of a unit cell $j$ of the photonic lattice composed of three waveguides $s_{j}\equiv A_{j}, B_{j}, C_{j}$ forming a triangle with central angle $\theta$. The distances between waveguide centers are $d_{A_{j}-B_{j}}=d_{A_{j}-C_{j}}\equiv d$, and $d_{B_{j}-C_{j}}=2d\sin(\theta/2)$. Each waveguide supports two $\mathcal{l=}1$ modes with positive and negative circulations. The coupling amplitudes between modes with equal circulations $c_{1,1}\equiv c_{1}$ are represented by the black solid arrows and the coupling amplitudes between modes with opposite circulations are ${c_{1,-1}\equiv c}_{2}$, represented by the blue dashed arrow, and $c_{1,-1}\equiv c_{2}e^{i2\phi_{0}}$, represented by the red dotted arrow, for the phase origin $\phi_{0}$fixed along the $A_{j}⟷C_{j}$ direction. The grey arrow indicates the coupling between $B_{j}$ and $C_{j}$ that can be neglected for $\theta>\pi/3$.

We consider a unit cell of the diamond chain configuration formed by three identical cylindrical waveguides $s_{j}\equiv A_{j},B_{j},C_{j}$ forming a triangle with central angle $\theta$ represented in Figure S1. Here, we focus on the subset of OAM modes with $\mathcal{l=}1$ topological charge. In this subset, the coupling amplitude between modes with equal circulations is given by $c_{1,1}\equiv c_{1}$ (black solid arrows in Figure S1), while the coupling amplitude between modes with opposite circulations is given by $c_{1,-1}\equiv c_{2}e^{i2\phi_{0}}$, with $\phi_{0}$ being the phase origin^1^. Specifically, we set $\phi_{0}$ along the $A_{j}⟷C_{j}$ direction such that the coupling amplitude is real in the $A_{j}⟷C_{j}$ direction and complex in the $A_{j}⟷B_{j}$ direction, as represented by the blue dashed and red dotted arrows in Figure S1, respectively. Moreover, the coupling between $B_{j}$ and $C_{j}$ can be neglected for $\theta>\pi/3$ since the coupling amplitudes decay exponentially with the distance. Note that, for the evanescently coupled waveguides with a small index contrast here employed, the spin-orbit interaction can be neglected and it does not affect the light dynamics^2^. Therefore, light dynamics can be described by coupled-mode equations of the form

$$i\frac{d}{dz}\boldsymbol{\Psi}\mathcal{=H}\boldsymbol{\Psi} \left( S1 \right)$$

where $\boldsymbol{\Psi}=\left( c_{j}^{+},c_{j}^{-},a_{j}^{+},a_{j}^{-},b_{j}^{+},b_{j}^{-} \right)^{T}$, with $a_{j}^{\pm}$, $b_{j}^{\pm}$ and $c_{j}^{\pm}$ being the modal field amplitudes of the $\mathcal{l=}1$ mode with positive and negative circulations in $A_{j}, B_{j}$ and $C_{j}$ waveguides, respectively, and the Hamiltonian is given by^3^

$$\mathcal{H}_{1}=\left( \begin{matrix} \beta_{C}^{+} & 0 & c_{1} & c_{2} & 0 & 0 \\ 0 & \beta_{C}^{-} & c_{2} & c_{1} & 0 & 0 \\ c_{1} & c_{2} & \beta_{A}^{+} & 0 & c_{1} & c_{2}e^{-i2\theta} \\ c_{2} & c_{1} & 0 & \beta_{A}^{-} & c_{2}e^{i2\theta} & c_{1} \\ 0 & 0 & c_{1} & c_{2}e^{-i2\theta} & \beta_{B}^{+} & 0 \\ 0 & 0 & c_{2}e^{i2\theta} & c_{1} & 0 & \beta_{B}^{-} \end{matrix} \right) (S2)$$

where $\beta_{s_{j}}^{\pm}$ is the propagation constant of mode $\mathcal{l=}1$ with positive and negative circulations in waveguide $s_{j}$. Note that $\beta_{s_{j}}^{+}=\beta_{s_{j}}^{-}$, and, since we consider identical waveguides, the diagonal elements can be factorized introducing a global phase into the dynamics. Moreover, imposing $\theta=\pi/2$, the coupled-mode equations (S1) read

$$i\frac{da_{j}^{\pm}}{dz}=\beta_{A_{j}}^{1}a_{j}^{\pm}+c_{1}\left( b_{j}^{\pm}+b_{j+1}^{\pm}+c_{j}^{\pm}+c_{j+1}^{\pm} \right)+c_{2}\left( b_{j+1}^{\mp}-b_{j}^{\mp}+c_{j}^{\mp}-c_{j+1}^{\mp} \right) (S3a)$$

$$i\frac{db_{j}^{\pm}}{dz}=\beta_{B_{j}}^{1}b_{j}^{\pm}+c_{1}\left( a_{j}^{\pm}+a_{j+1}^{\pm} \right)+c_{2}\left( {a_{j+1}^{\mp}-a}_{j}^{\mp} \right) (S3b)$$

$$i\frac{dc_{j}^{\pm}}{dz}=\beta_{B_{j}}^{1}c_{j}^{\pm}+c_{1}\left( a_{j}^{\pm}+a_{j+1}^{\pm} \right)+c_{2}\left( a_{j}^{\mp}-a_{j+1}^{\mp} \right) (S3c)$$

where $a_{j}^{\pm}$, $b_{j}^{\pm}$ and $c_{j}^{\pm}$ being the modal field amplitudes of the $\mathcal{l=}1$ mode with positive and negative circulations in $A_{j}, B_{j}$ and $C_{j}$ waveguides, respectively.

Finally, let us momentarily consider a unit cell with the in-line configuration $(\theta=\pi)$ to calculate the coupling strengths, $c_{1}$ and $c_{2}$, in a very convenient way. To this aim, one needs to perform a basis rotation into symmetric and antisymmetric supermodes of the form

$$\left| S_{j}^{S} \right\rangle=\frac{1}{\sqrt{2}}\left( \left| S_{j}^{+} \right\rangle+ \left| S_{j}^{-} \right\rangle\right) \text{and} \left| S_{j}^{A} \right\rangle=\frac{1}{\sqrt{2}}\left( \left| S_{j}^{+} \right\rangle- \left| S_{j}^{-} \right\rangle\right) (S4)$$

where $\left| S_{j}^{\pm} \right\rangle$ accounts for the mode with positive or negative circulation propagating in waveguide *j*. Therefore, in this configuration, the symmetric $\left| A_{j}^{S} \right\rangle$ and antisymmetric $\left| A_{j}^{A} \right\rangle$ supermodes in the central waveguide read

$$\left| A_{j}^{S} \right\rangle=\frac{1}{\sqrt{2}}\left( \left| A_{j}^{+} \right\rangle+ \left| A_{j}^{-} \right\rangle\right) \text{and} \left| A_{j}^{A} \right\rangle=\frac{1}{\sqrt{2}} \left( \left| A_{j}^{+} \right\rangle- \left| A_{j}^{-} \right\rangle\right) (S5)$$

and it is straightforward to check that they are only coupled to^3^

$$\left| {K_{j}}^{S} \right\rangle=\frac{1}{\sqrt{2}}\left( \left| C_{j}^{S} \right\rangle+\left| B_{j}^{S} \right\rangle\right) \text{and} \left| {K_{j}}^{A} \right\rangle=\frac{1}{\sqrt{2}}\left( \left| C_{j}^{A} \right\rangle+\left| B_{j}^{A} \right\rangle\right) (S6)$$

with coupling strengths $c_{S}=\sqrt{2}\left( c_{1}+c_{2} \right)$ and $c_{A}=\sqrt{2}\left| c_{1}-c_{2} \right|$, respectively. Therefore, by injecting the symmetric supermode in waveguide $A_{j}$, one can measure the beating length $L_{S}=\pi/{2c_{S}}$ as it can be observed in Fig. S2a. On the other hand, by injecting the antisymmetric supermode in waveguide $A_{j}$, one can measure the beating length $L_{A}=\pi/{2c_{A}}$ as it can be seen in Fig. S2b. From $L_{S}$ and $L_{A}$ the dependence of the $c_{1}$ and $c_{2}$ with respect to the distance $d$ between waveguides can be characterized, see Table S1. Note that, the fact that $\left| c_{2} \right|>|c_{1}|$ can be intuitively understood by realizing that the phases between modes propagating with opposite (equal) circulations flow in the same (opposite) direction at the region between waveguides where the overlap between modes occurs^3^.

| $\boldsymbol{d}\mathbf{(}\text{μm}\mathbf{)}$ | $\boldsymbol{L}_{\mathbf{A}}\mathbf{(}\text{mm}\mathbf{)}$ | $\boldsymbol{c}_{\boldsymbol{A}}\mathbf{(}\text{mm}^{\mathbf{-1}}\mathbf{)}$ | $\boldsymbol{L}_{\mathbf{S}}\mathbf{(}\text{mm}\mathbf{)}$ | $\boldsymbol{c}_{\mathbf{S}}\mathbf{(}\text{mm}^{\mathbf{-1}}\mathbf{)}$ | $\boldsymbol{c}_{\mathbf{1}}\mathbf{(}\text{mm}^{\mathbf{-1}}\mathbf{)}$ | $\boldsymbol{c}_{\mathbf{2}}\mathbf{(}\text{mm}^{\mathbf{-1}}\mathbf{)}$ |
| --- | --- | --- | --- | --- | --- | --- |
| 5.0 | 0.25 | 6.28 | 0.74 | 2.12 | 1.47 | 2.97 |
| 5.5 | 0.34 | 4.62 | 1.05 | 1.50 | 1.10 | 2.16 |
| 6.0 | 0.48 | 3.31 | 1.53 | 1.03 | 0.81 | 1.53 |
| 6.5 | 0.62 | 2.55 | 2.10 | 0.75 | 0.64 | 1.17 |
| 7.0 | 0.79 | 2.00 | 2.90 | 0.54 | 0.52 | 0.90 |
| 7.5 | 1.05 | 1.50 | 4.05 | 0.39 | 0.39 | 0.67 |

**Table S1.** Coupling strengths $c_{S}$, $c_{A}$, $c_{1}$ and $c_{2}$, and beating lengths $L_{S}$ and $L_{A}$ for different separation distances $d$ between waveguides.

**
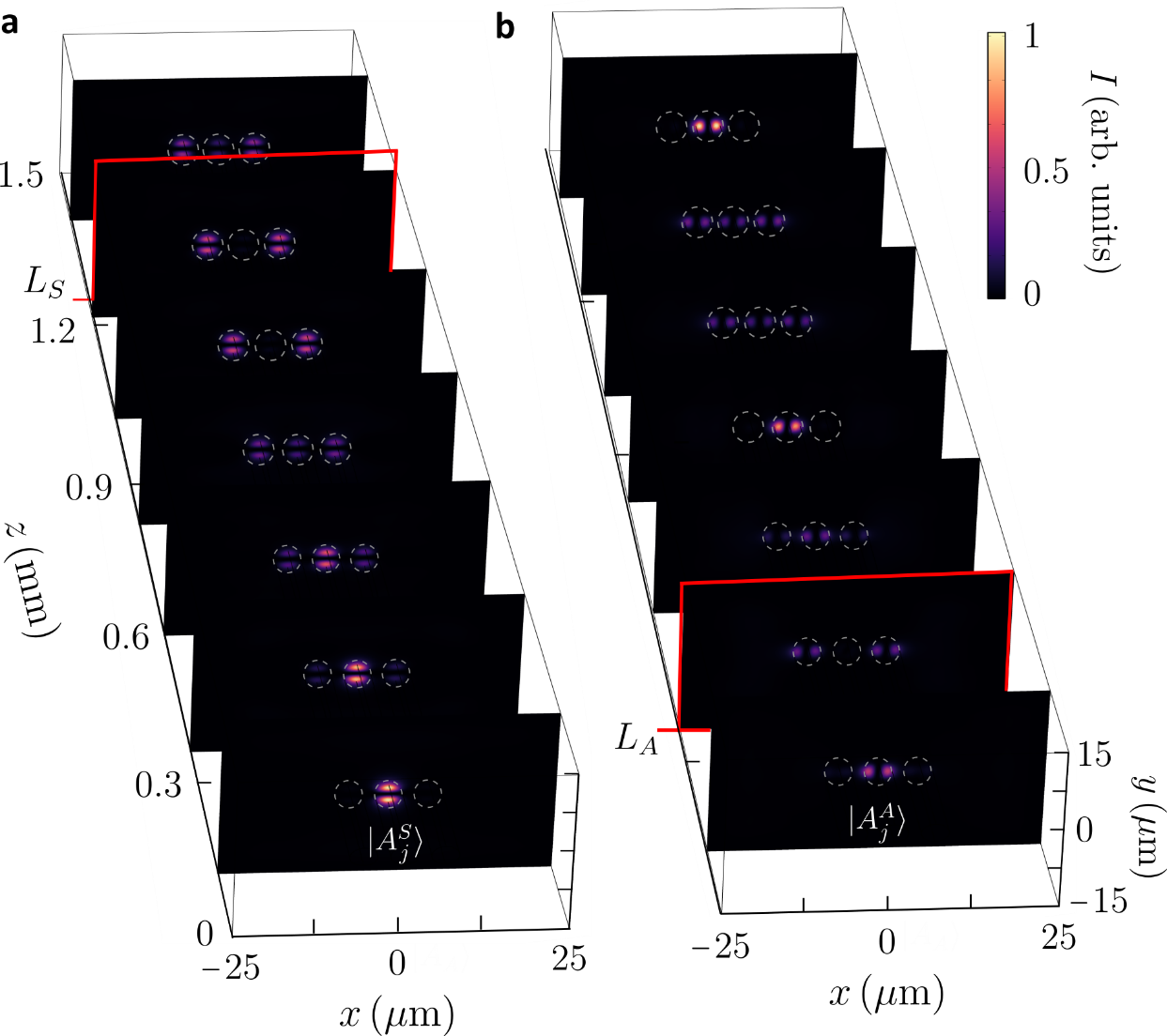
**

**Fig. S2. Coupling characterization.** Numerically simulated light evolution along the propagation direction when we inject **a** the symmetric supermode in the central waveguide and **b** the antisymmetric supermode in the central waveguide of three waveguides in line. The parameters used in the simulations are $d=5.5 \text{μm}$, $R=1.9 \text{μm}$, $n_{\mathrm{core}}=1.548$, $n_{\mathrm{clad}}=1.540$ and $\lambda_{0}=700 \text{nm}$. Moreover, $L_{S}$ and $L_{A}$ correspond to the coupling length of the symmetric and antisymmetric supermodes, respectively.

**Supplementary II: Nearest neighbor vs. next-nearest neighbor couplings**

**
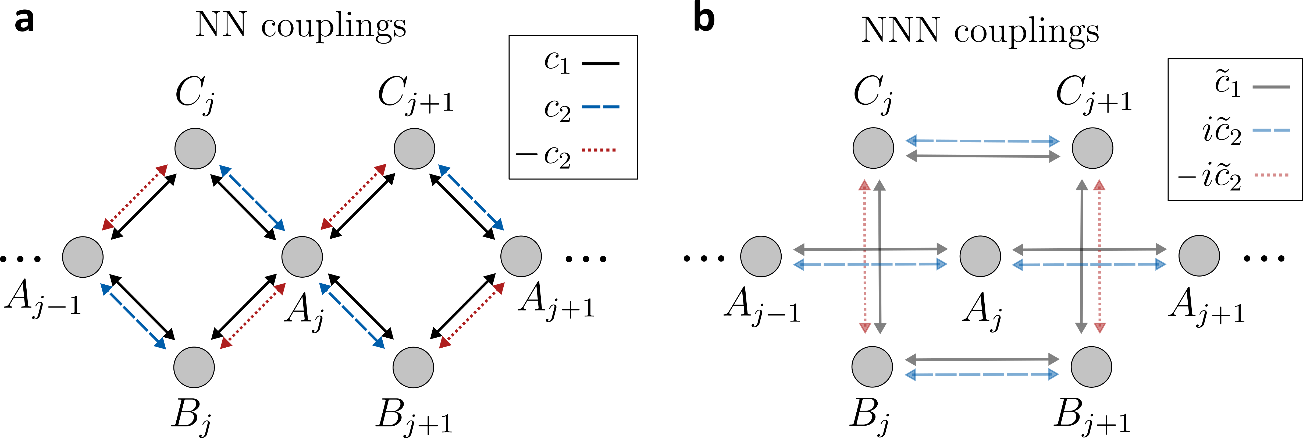
**

**Fig. S3. Couplings for** $\mathcal{l=}\boldsymbol{1}$ **modes.** Schematic representation of the **a** nearest-neighbor (NN) couplings between $\mathcal{l=}1$ modes with equal $c_{1,1}=c_{1}$ and opposite ${c_{1,-1}=\pm c}_{2}$ circulations, represented by the black solid, blue dashed and red dotted arrows, respectively, and **b** next-nearest neighbor (NNN) couplings between $\mathcal{l=}1$ modes with equal $\tilde{c}_{1,1}=\tilde{c}_{1}$ and opposite ${\tilde{c}_{1,-1}=\pm i\tilde{c}}_{2}$ circulations, represented by the light black solid, light blue dashed and light red dotted arrows, respectively. Note that the angle between the $C_{j}\leftrightarrow A_{j}$ and $B_{j}\leftrightarrow A_{j}$ lines is $\theta=\pi/2$.

Considering a diamond-chain formed by $N$ unit cells with $\theta=\pi/2$ (see Fig. S1), the nearest neighbor (NN) couplings occur between $A_{j}\leftrightarrow B_{j}$*,* $A_{j}\leftrightarrow B_{j+1}$*,* $A_{j}\leftrightarrow C_{j}$ and $A_{j}\leftrightarrow C_{j+1}$, as illustrated in Fig. S3a, while the next-nearest neighbor (NNN) couplings occur between $A_{j}\leftrightarrow A_{j\pm1}$*,* $B_{j}\leftrightarrow B_{j\pm1}$*,* $C_{j}\leftrightarrow C_{j\pm1}$ and $B_{j}\leftrightarrow C_{j}$ as illustrated in Fig. S3b. Moreover, although the coupling strengths $c_{1}$ and $c_{2}$ between NN waveguides depend on the separation between waveguides, *d*, illustrated in Fig. 1c of the main text, the coupling strengths $\tilde{c}_{1}$ and $\tilde{c}_{2}$ between NNN waveguides cannot be directly retrieved from the values corresponding to $\sqrt{2}d$ in Fig. 1c. The reason is that the NN waveguide lying between the NNN waveguides has to be taken into account when calculating the overlap integral between the modes, reducing the effective NNN coupling strengths^4^, which are $\sim10$ times weaker than the NN ones in our case. Therefore, the NNN couplings can be safely neglected. This fact can be easily verified by comparing the light dynamics described by the coupled-mode (CM) equations (S3) considering only NN couplings (see Fig. S4a) or also including NNN couplings (see Fig. S4b), with the numerical simulations performed using finite-difference methods (FDM) that take into account all the possible couplings. Specifically, we can observe in Fig. S4 how NN couplings already give a very good approximation of light dynamics, validating our assumption.

**
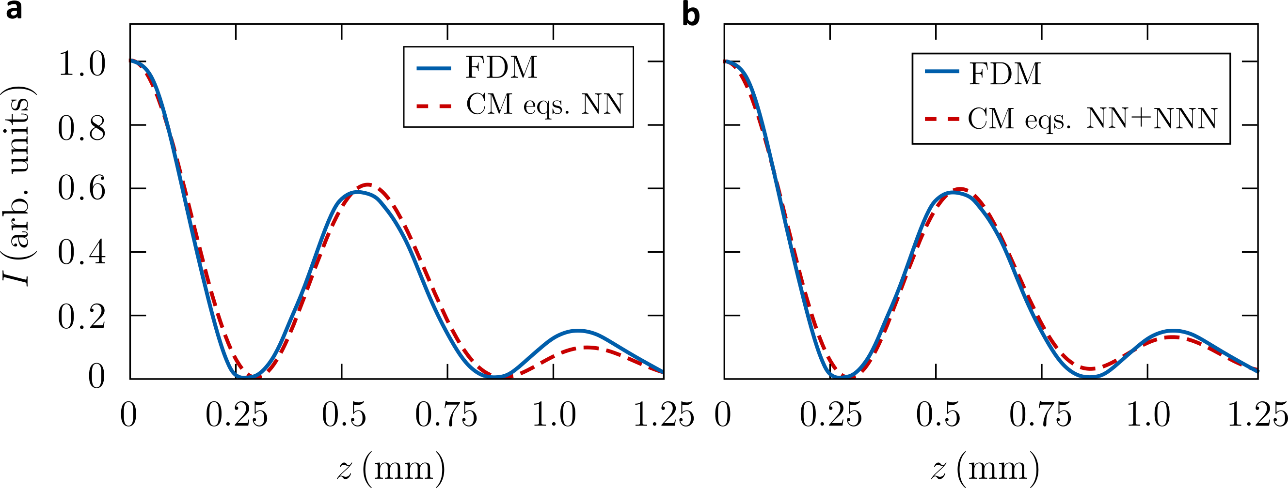
**

**Fig. S4. Coupled-mode equations simulations.** Numerically calculated intensity propagating in waveguide $A_{4}$ when the $\mathcal{l=}1$ mode is injected in waveguide $A_{4}$. The blue solid lines correspond to the full numerical simulations performed using finite difference method (FDM) calculations that take into account all couplings while the red dashed lines correspond to the coupled-mode (CM) equations considering **a** NN couplings, **b** NN and NNN couplings. All the simulations were performed using $d=5.3 \text{μm}$, $R=1.9 \text{μm}$, $n_{\mathrm{core}}=1.548$, $n_{\mathrm{clad}}=1.540$ and $\lambda_{0}=700 \text{nm}$.

**Supplementary III: Basis rotations for** $\mathcal{l=}\boldsymbol{1}$ **modes**

**
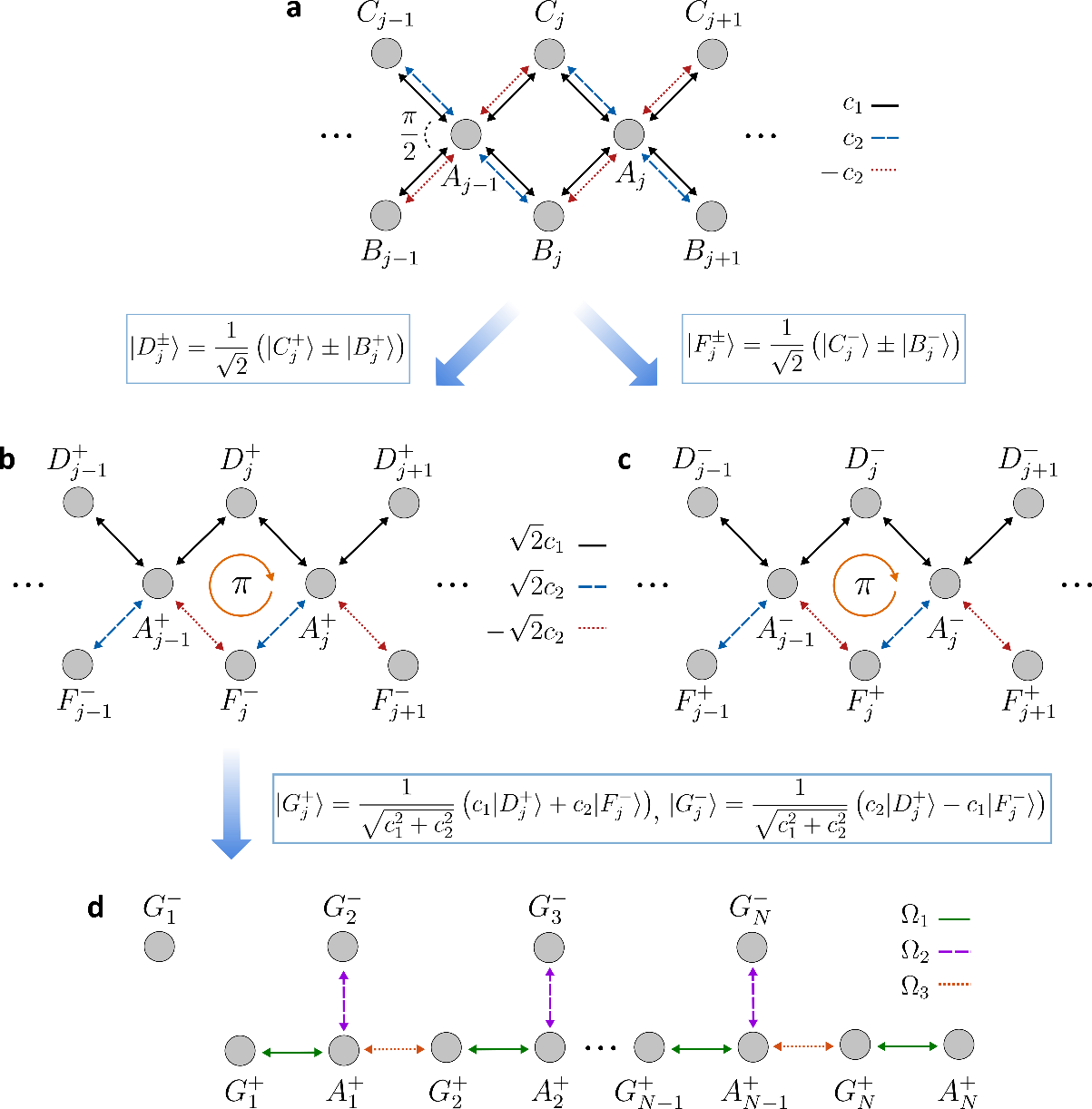
**

**Fig. S5. Basis rotations. a** Schematic representation of the photonic lattice arranged in a diamond chain configuration with central angle $\theta=\pi/2$ supporting two $\mathcal{l=}1$ modes per waveguide. The coupling amplitudes between modes with equal and opposite circulations are given by $c_{1,1}=c_{1}$ and ${c_{1,-1}=\pm c}_{2}$, represented by the black solid, blue dashed and red dotted arrows, respectively. **b** and **c** schematic representation of the decoupled diamond chain lattices obtained performing a basis rotation into $\left. |D_{j}^{\pm} \right\rangle$ and $\left. |F_{j}^{\pm} \right\rangle$. In this new basis there is only one mode per waveguide and a $\pi$-flux is present in the plaquettes. The coupling amplitudes $\sqrt{2}c_{1}, \sqrt{2}c_{2}$ and ${-\sqrt{2}c}_{2}$ are represented by the black solid, blue dashed and red dotted arrows, respectively. **d** Schematic representation of the modified SSH chain obtained performing a second basis rotation for the positive circulation sub-chain into $\left. |G_{j}^{\pm} \right\rangle$. The coupling amplitudes $\Omega_{1}\equiv\sqrt{2}\sqrt{c_{1}^{2}+c_{2}^{2}}$, $\Omega_{2}\equiv2\sqrt{2}c_{1}c_{2}/\sqrt{c_{1}^{2}+c_{2}^{2}}$ and $\Omega_{3}\equiv\sqrt{2}(c_{1}^{2}-c_{2}^{2})/\sqrt{c_{1}^{2}+c_{2}^{2}}$ are represented by the green solid, purple dashed and orange dotted arrows, respectively.

Let us consider the diamond chain for the $\mathcal{l=}1$ manifold with coupling strengths $c_{1}, c_{2}$ and ${-c}_{2}$, represented by the black solid, blue dashed and red dotted arrows in Figure S2a, respectively. By performing the basis rotation^5^

$$\left| D_{j}^{\pm} \right\rangle=\frac{1}{\sqrt{2}}\left( \left| C_{j}^{+} \right\rangle\pm\left| B_{j}^{+} \right\rangle\right) \text{and} \left| F_{j}^{\pm} \right\rangle=\frac{1}{\sqrt{2}}\left( \left| C_{j}^{-} \right\rangle\pm\left| B_{j}^{-} \right\rangle\right) (S7)$$

the original chain with two OAM modes with positive and negative circulations per waveguide splits into two identical and decoupled sub-chains depicted in Figures S2b and S2c. In the first (second) sub-chain, sustaining one mode per waveguide, the $\left| D_{j}^{+} \right\rangle$ and $\left| F_{j}^{-} \right\rangle$ $\left( \left| D_{j}^{-} \right\rangle\mathrm{and}\left| F_{j}^{+} \right\rangle\right)$ supermodes are coupled to the ${|A}_{j}^{+}\rangle$ $\left( {|A}_{j}^{-}\rangle\right)$ mode with coupling strengths $\sqrt{2}c_{1}, \sqrt{2}c_{2}$ and ${-\sqrt{2}c}_{2}$, represented by the black solid, blue dashed and red dotted arrows in Figure S2b(c). This mapping allows to explain the degeneracy of the energy bands of the original structure, discussed in the main text, and the gap opening through the existence of a $\pi$-flux in the plaquettes. Moreover, in order to get more insight into the topology of the system and the origin of the non-zero energy flat-bands, one can perform a second basis rotation^5^

$$\left| G_{j}^{+} \right\rangle=\frac{1}{\sqrt{c_{1}^{2}+c_{2}^{2}}}\left( c_{1}\left| D_{j}^{+} \right\rangle+c_{2}\left| F_{j}^{-} \right\rangle\right) \text{and} \left| G_{j}^{-} \right\rangle=\frac{1}{\sqrt{c_{1}^{2}+c_{2}^{2}}}\left( c_{2}\left| D_{j}^{+} \right\rangle-c_{1}\left| F_{j}^{-} \right\rangle\right) (S8)$$

By doing so, the sub-chain of Figure S2b can be mapped into a modified SSH chain with alternating strong $\left( \Omega_{1}\equiv\sqrt{2}\sqrt{c_{1}^{2}+c_{2}^{2}} \right)$ and weak $\left( \Omega_{3}\equiv\sqrt{2}(c_{1}^{2}-c_{2}^{2})/\sqrt{c_{1}^{2}+c_{2}^{2}} \right)$ couplings and extra dangling states coupled by $\Omega_{2}\equiv2\sqrt{2}c_{1}c_{2}/\sqrt{c_{1}^{2}+c_{2}^{2}}$, represented by the green solid, purple dashed and orange dotted arrows in Figure S2d, respectively. Note that a similar derivation can be made for the chain of Figure S2c by substituting $F_{j}\leftrightarrow D_{j}$ in Eq. (S8). In the ${c_{2}}/{c_{1}\to1}$ limit, $\Omega_{3}\to0$, hence, the chain of Figure S2d is decoupled into trimers whose (non-zero energy) eigenmodes read^5^

$$\left| E_{j,1}^{\pm} \right\rangle=\frac{1}{2} \left( \left| G_{j}^{+} \right\rangle\pm\sqrt{2} \left| A_{j}^{+} \right\rangle+\left| G_{j+1}^{-} \right\rangle\right) (S9)$$

which can be rewritten in the original basis as

$$\left| E_{j,1}^{\pm} \right\rangle=\frac{1}{4}\left[ \left| C_{j}^{+} \right\rangle+\left| B_{j}^{+} \right\rangle+\left| C_{j+1}^{+} \right\rangle+\left| B_{j+1}^{+} \right\rangle+\left| C_{j}^{-} \right\rangle\right.\left. -\left| B_{j}^{-} \right\rangle-\left| C_{j+1}^{-} \right\rangle+|B_{j+1}^{-}\rangle\right]\pm\frac{1}{\sqrt{2}}\left| A_{j}^{+} \right\rangle(S10)$$

A similar expression $\left| E_{j,2}^{\pm} \right\rangle$ can be obtained for the chain represented in Figure S2c just by changing the positive circulations of Eq. (S10) by negative circulations and vice versa. These non-zero energy flat-band modes are localized in the $j$ and $j+1$ unit cells and allow to express $\mathcal{l=}1$ OAM modes with positive and negative circulations in a central waveguide $A_{j}$ of the lattice as

$\left| A_{j}^{+} \right\rangle=\frac{1}{\sqrt{2}}\left( \left| E_{j,1}^{+} \right\rangle-\left| E_{j,1}^{-} \right\rangle\right) \text{and} \left| A_{j}^{-} \right\rangle=\frac{1}{\sqrt{2}}\left( \left| E_{j,2}^{+} \right\rangle-\left| E_{j,2}^{-} \right\rangle\right) (S11)$

respectively. Besides, since $\left| A_{j}^{+} \right\rangle$ and $\left| A_{j}^{-} \right\rangle$belong to different decoupled sub-chains, any superposition of $\mathcal{l=}1$ OAM modes with positive and negative circulations injected in $A_{j}$ will be trapped in the cage formed by $A_{j}, B_{j},C_{j},B_{j+1}, C_{j+1}$ as it evolves along the propagation direction, producing the Aharonov-Bohm caging effect.

**Supplementary IV: Complementary results of the Aharonov-Bohm caging effect**

**
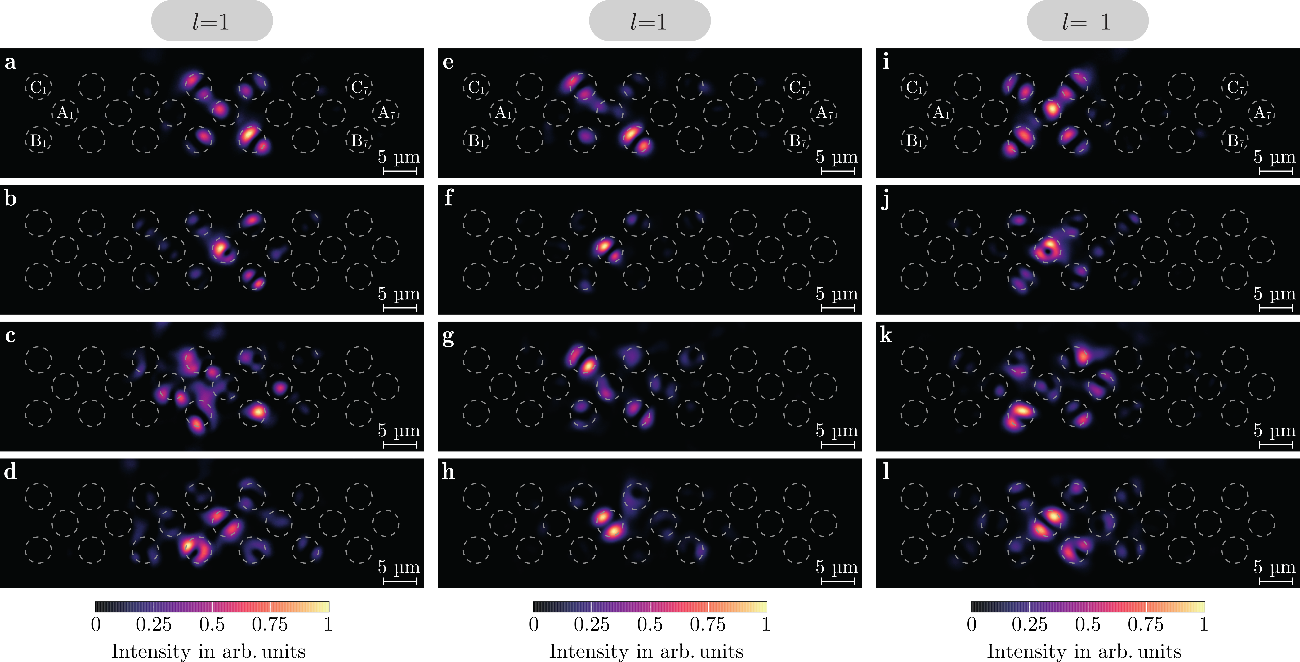
**

**Fig. S6. Complementary results of the Aharonov-Bohm caging effect.** Experimentally observed output intensities, obtained by exciting (left column) waveguide $A_{4}$ using the OAM mode with $\mathcal{l=}1$ and positive circulation, at **a** $z\boldsymbol{=}250 \mu m$, **b** $z=500 \mu m$, **c** $z=750 \mu m$ and **d** $z=1000 \mu m$, (central column) waveguide $A_{3}$ using the OAM mode with $\mathcal{l=}1$ and positive circulation, at **e** $z=250 \mu m$, **f** $z=500 \mu m$, **g** $z=750 \mu m$ and **h** $z=1000 \mu m$, (right column) waveguide $A_{3}$ using the OAM mode with $\mathcal{l=}1$ and negative circulation at **i** $z=250 \mu m$, **j** $z=500 \mu m$, **k** $z=750 \mu m$ and **l** $z=1000 \mu m$. The diamond chain lattice is composed of $7$ unit cells, i.e., 21 waveguides with radius $R=1.9 \text{μm}$ and nearest-neighbor separation $d=5.5 \mu m$. The wavelength used is $\lambda_{0}=700 \text{nm}$. The intensity distribution in each of the figures is normalized to the maximum intensity value of the corresponding figure.

In addition to the different experimental results showing the AB caging effect in Fig. S6, we also compare the experimental results obtained by exciting waveguide $A_{4}$ with the $\mathcal{l=}1$ mode and negative circulation (see Fig. S7a-e) with numerical simulations (see Fig. S7f-o). In particular considering perfectly cylindrical waveguides, we can observe how in the first recombination the mode still looks like a donut mode in the simulations (see Fig. S7h), rather than the lobe-shaped mode that seems to appear in the experiment (see Fig. S7c), while in the second recombination we obtain the lobe-shaped mode in both cases (see Fig. S7e and j). The changing from the donut mode into the lobe-shaped mode in the lattice is due to the non-symmetric presence of the NNN waveguides (they are present to the left and right of the chain, but not to the top and bottom), which slightly breaks the degeneracy of the modes. Moreover, the faster appearance of the lobe-shaped mode in the experiment (see Fig. S7a-e) with respect to the simulations (Fig. S7f-j) is due to a slight ellipticity of the fabricated waveguides. This has been checked by sending a donut mode into a single waveguide without neighbors and observing that the mode is also transformed into a lobe-shaped mode, which rotates when rotating the sample. Introducing this ellipticity in the diamond-chain lattice, we can observe how the lobe-shaped mode is obtained faster (see Fig. S7k-o), in better agreement with the experimental results of Fig. S7a-e. This confirms that our experimental measurements are influenced by the slight ellipticity of the waveguides.


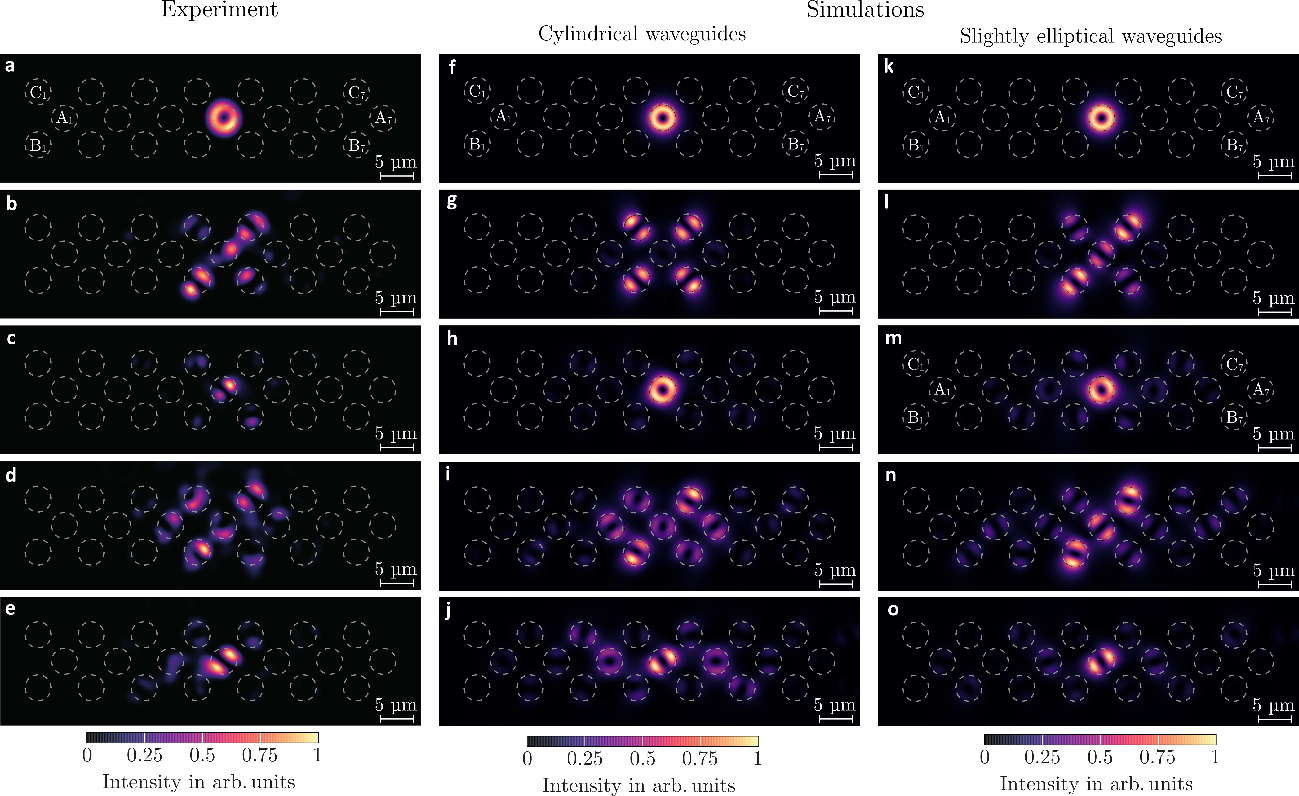


**Fig. S7.** **Experimental vs. simulated results of the Aharonov-Bohm caging effect.** Experimentally observed input and output intensities, obtained by exciting waveguide $A_{4}$ using the OAM mode with $\mathcal{l=}1$ and negative circulation, at **a** $z\boldsymbol{=}0 \mu m$, **b** $z=250 \mu m$, **c** $z=500 \mu m$, **d** $z=750 \mu m$ and **e** $z=1000 \mu m$. Input and output intensities numerically obtained by exciting waveguide $A_{4}$ using the OAM mode with $\mathcal{l=}1$ and negative circulation considering cylindrical waveguides at **f** $z=0 \mu m$, **g** $z=250 \mu m$, **h** $z=500 \mu m$, **i** $z=750 \mu m$ and **j** $z=1000 \mu m$, and considering slightly elliptical waveguides at **k** $z=0 \mu m$, **l** $z=250 \mu m$, **m** $z=500 \mu m$, **n** $z=750 \mu m$ and **o** $z=1000 \mu m$. The diamond chain lattice is composed of $7$ unit cells, i.e., 21 waveguides with radius $R=1.9 \text{μm}$ and nearest-neighbor separation $d_{\exp}=5.5 \mu m$. The wavelength used is $\lambda_{0}=700 \text{nm}$. The experimental results are in agreement with the simulations once a small correction of $\Delta d=d_{\exp}-d_{\mathrm{sim}}=0.2 \mu m$ is introduced. Moreover, in **k**-**o** the waveguides are slightly elliptical, i.e. the diameter of the waveguide is slightly bigger in the $y$- direction than in the $x$- direction by $\Delta R=0.1 \mu m$. The intensity distribution is normalized to the maximum intensity value of the corresponding figure.

**Supplementary V: Robustness of the Aharonov-Bohm caging effect**

To test the robustness of the AB-caging effect, we have numerically analyzed the deviations induced by changes in the separation between waveguides $d$ and the refractive index contrast $\Delta n=n_{\mathrm{core}}-n_{\mathrm{clad}}$. In this vein we have analyzed two situations (i) uniform deviations and (ii) random deviations, of the distances and the refractive index contrast.

1. Uniform deviations

Small deviations in the separation between waveguides induce small changes in the positions and values of the minima and maxima of the caging. For instance, if the separation between waveguide centers varies by $\Delta d=\pm0.2 \mu m$ as considered in Fig. S8a, the position of the first maximum $z_{1}$ is displaced by $\Delta z_{1}=\pm0.04 \text{mm}$ and its value $I_{1}$ changes by $\Delta I_{1}=\pm0.03$. The reason why the effect is robust against these variations relies on the fact that $c_{2}/c_{1}$ only changes by $\pm0.05$, and thereby, the band structure remains almost unaltered. Moreover, uniform variations in the refractive index contrast have a similar effect and the AB caging effect remains robust. In particular, we can observe in Fig. S8b how for $\Delta n=\pm0.001$ deviations, the first maximum is displaced by $\Delta z_{1}=\pm0.05 \text{mm}$ and its value changes by $\Delta I_{1}=\pm0.09$. Note that, the larger decrease for $n_{\mathrm{core}}=1.547$ occurs because $\beta_{1}/k_{0}\approx k_{0}n_{\mathrm{clad}}$ inducing losses, as the mode is only weakly bound to the waveguide.

1. Random deviations

Small random deviations of the distances between waveguides induce similar changes in the positions and values of the maxima of intensity in the central waveguide as uniform deviations. In particular, as illustrated in Fig. S8c, for an average of five different random deviations $d_{j}\pm0.2 \mu m$ in the $x$- and $y$- directions, the position of the first maximum $z_{1}$ is displaced by $\Delta z_{1}=\pm0.03 \text{mm}$ and its peak value $I_{1}$ changes by $\Delta I_{1}=\pm0.03$. Therefore, the effect is still robust against random distance variations. On the other hand, the effect is no longer robust against random refractive index contrast deviations because different $\Delta n_{j}$ imply different propagation constants $\beta_{1,j}\neq\beta_{1,i}$, where $i$ and $j$ account for two different waveguides, decreasing the coupling between modes. In particular, as illustrated in Fig. S8d, we can see how for an average of five different random deviations $n_{core,j}\pm0.001$, the first maximum displaces its position $z_{1}$ by a similar value than before, $\Delta z_{1}=\pm0.04 \text{mm}$, and its value $I_{1}$ changes by $\Delta I_{1}=\pm0.1$. However, one can observe how after the first peak one does not reach the zero minimum due to detuning between waveguides and the second oscillation is clearly different from the case without disorder. Therefore, the system is sensitive to random variations in the refractive index contrast. For this reason, we have restricted our experimental observations to the central waveguides ($A_{3}$ and $A_{4}$) where the deviations in the index contrast due to fabrication are the lowest ones.


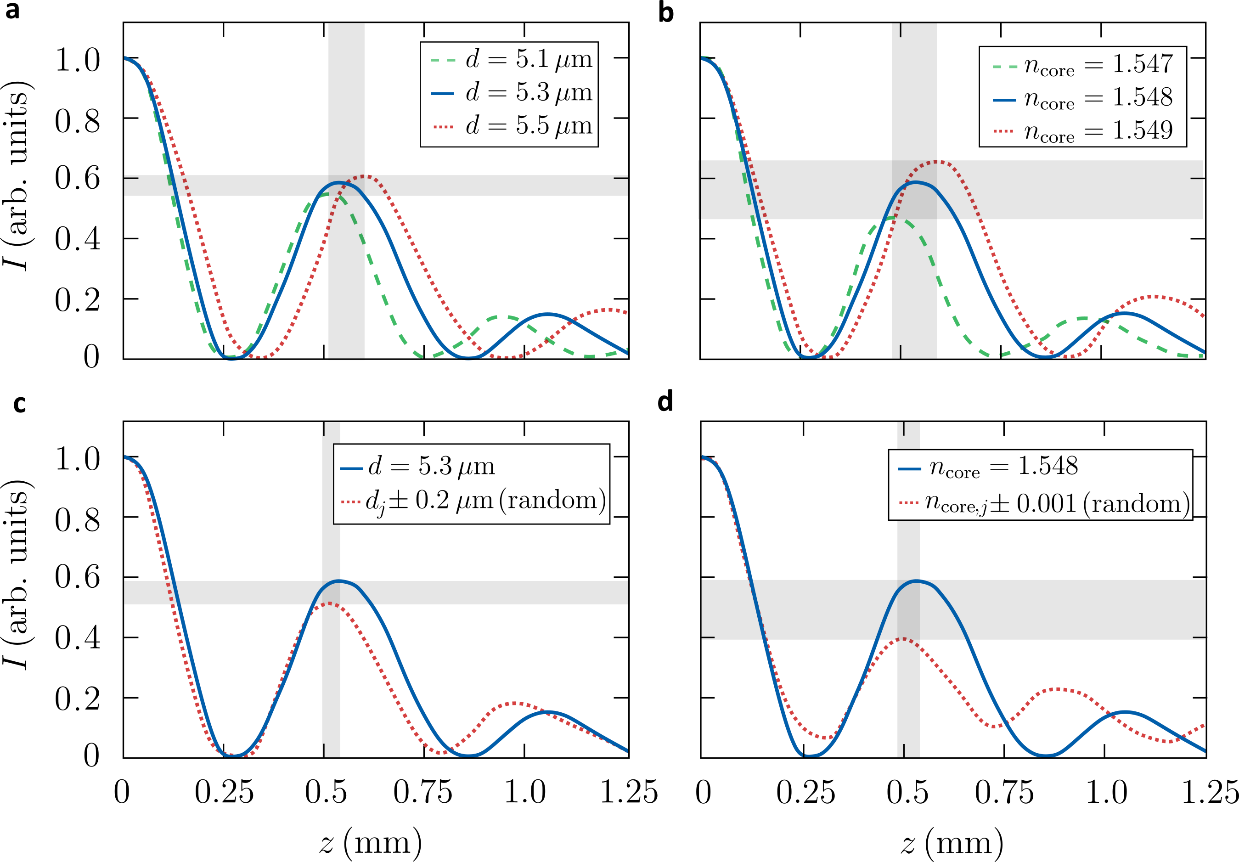


**Fig. S8. Robustness of the AB caging effect.** Numerically calculated intensity propagating in waveguide $A_{4}$ using finite-difference methods for **a** $d=5.1 \text{μm}$ (green dashed line), $d=5.3 \text{μm}$ (blue solid line) and $d=5.5 \text{μm}$ (red dotted line) and $n_{\mathrm{core}}=1.548$, **b** $n_{\mathrm{core}}=1.547$ (green dashed line), $n_{\mathrm{core}}=1.548$ (blue solid line) and $n_{\mathrm{core}}=1.549$ (red dotted line) and$d=5.3 \text{μm}$, **c** $d=5.3 \text{μm}$ (blue solid line) and an average of five random variations of $d_{j}\pm0.2 \text{μm}$ (red dotted line) and $n_{\mathrm{core}}=1.548$, **d** $n_{\mathrm{core}}=1.548$ (blue solid line) and an average of five random variations of $n_{\mathrm{core},j}\pm0.001$ (red dotted line), and$d=5.3 \text{μm}$. The rest of the parameters are $R=1.9 \text{μm}$, $n_{\mathrm{clad}}=1.540$ and $\lambda_{0}=700 \text{nm}$. The vertical and horizontal shaded regions indicate the deviations with respect to the position of the first maximum $\Delta z_{1}$ and its value $\Delta I_{1}$, respectively.

**Supplementary VI: Experimental measurement set-up**

**
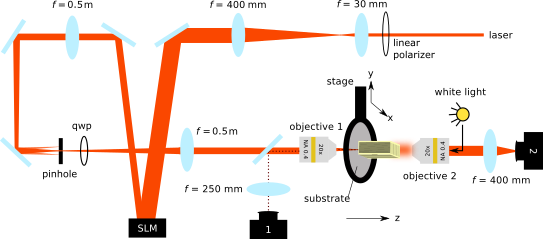
**

**Fig. S9. Experimental set-up for the measurements.** Laser light from a white light laser (NKT photonics) is sent through a VARIA filter box to select a wavelength of 700 nm. The beam is linearly polarized, expanded and sent onto a spatial light modulator (SLM). We load a hologram onto the SLM that consists of a phase-only vortex, with an added blazed grating to shift the pattern to the first diffraction order. Other orders are blocked by a pinhole. The beam is circularly polarized by a quarter wave-plate and imaged onto objective lens 1, which Fourier transforms the phase hologram to create a donut-shaped intensity profile with $\mathcal{l=}1$ and positive/negative circulations, or a Gaussian-shaped intensity profile with $\mathcal{l=}0$ and constant phase (depending on the hologram that we load). The reflection of the input mode is imaged via a beamsplitter onto camera 1. Using white light from a common torch lamp allows to additionally image the sample input facet onto camera 1 at the same time, to overlay the input mode with the waveguide position. The sample can be moved in the *x*- and *y*-directions by linear actuators (Zaber, smallest realistic step size approximately 100 nm). The output intensity at the sample output facet is imaged by objective lens 2 onto camera 2.

**Supplementary references**

1. Polo, J., Mompart, J. & Ahufinger, V. Geometrically induced complex tunnelings for ultracold atoms carrying orbital angular momentum. *Physical Review A* **93**, 033613 (2016).

2. Alexeyev, C. N. *et al*. Effect of the spin–orbit interaction on polarization conversion in coupled waveguides. *Journal of Optics A: Pure and Applied Optics* **11**, 125404 (2009).

3. Turpin, A. *et al*. Engineering of orbital angular momentum supermodes in coupled optical waveguides. *Scientific Reports* **7**, 44057 (2017).

4. Keil, R. *et al.* Direct measurement of second-order coupling in a waveguide lattice. *Applied Physics Letters* **107**, 241104 (2015).

5. Pelegrí, G. *et al.* Topological edge states with ultracold atoms carrying orbital angular momentum in a diamond chain. *Physical Review A* **99**, 023612 (2019).
